# Irregular, narrow-complex tachycardia

**Published:** 2018

**Authors:** Hoevelmann Julian, Viljoen Charle, Chin Ashley

**Affiliations:** Hatter Institute for Cardiovascular Research in Africa, University of Cape Town, South Africa, and Hannover Medical School, Department of Cardiology and Angiology, Hannover, Germany; Division of Cardiology, Groote Schuur Hospital and University of Cape Town, South Africa; Division of Cardiology, Groote Schuur Hospital and University of Cape Town, South Africa

**Keywords:** ECG, atrial fibrillation, atrial flutter, multifocal atrial tachycardia

## Abstract

The correct differentiation of an irregular, narrow-complex tachycardia has crucial implications for the therapeutic management of these conditions. In this article we present a differential diagnostic and treatment approach to irregular, narrow-complex tachycardias.

A 54-year-old lady with longstanding hypertension and a recent diagnosis of atrial fibrillation presented to the emergency unit with a two-day history of palpitations and mild dizziness. This was preceded by a few weeks of pedal oedema, with progressively worsening dyspnoea and effort intolerance. Clinically, she was mildly distressed with peripheral oedema, a respiratory rate of 24 breaths a minute and blood pressure of 129/84 mmHg. She had a low-volume pulse that was irregularly irregular, with a rate of around 120 beats per minute. The jugular venous pressure was not elevated, but her apex beat was diffuse and minimally displaced. On auscultation she had heart sounds of variable intensity, but no murmurs. Her chest had soft crackles in the bases.

Echocardiography revealed a non-dilated left ventricle with signs of concentric left ventricular hypertrophy (LVH) and impaired left ventricular (LV) function [LV ejection fraction (LVEF) of 40%]. The left atrium was dilated and there was mild mitral regurgitation (MR). She had normal pulmonary artery pressures and right ventricular (RV) function.

Her ECG (Fig. 1) showed an irregular, narrow-complex tachycardia. Very rapid, continuous and variable atrial activity was seen. The question was raised whether this ECG could be in keeping with ‘course’ atrial fibrillation or atrial flutter with variable block (Fig. 2). Careful inspection of the atrial activity revealed that there was subtle variation in rate, amplitude and morphology. Also, there is no pattern to the irregularity of the RR intervals. Based on these findings, the diagnosis of atrial fibrillation was made.

**Fig. 1:**
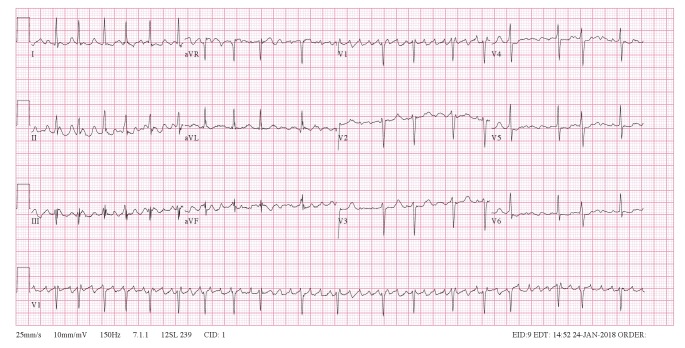
The 12-lead ECG with coarse atrial fibrillation, which could easily be mistaken for atrial flutter

**Fig. 2:**
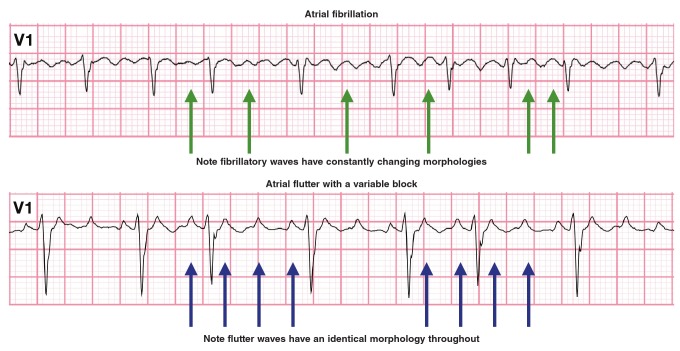
Comparison of coarse atrial fibrillation (fibrillatory wave morphology is not regular and uniform) and atrial flutter (flutter-wave morphology is regular and uniform).

The patient’s heart failure therapy was optimised with diuretics. She was initially treated with a rate-control strategy and oral anticoagulation in accordance with standard international guidelines.^1^,^2^ She will be seen in the rhythm clinic for consideration of a cardioversion and a future rhythm-control strategy with catheter ablation.

## Causes of irregular, narrow-complex tachycardia

The differential diagnosis of an irregular, narrow-complex tachycardia includes atrial fibrillation, atrial flutter with variable atrio-ventricular (AV) block and multifocal atrial tachycardia. Table 1 shows the differentiating electrocardiographic features.

Atrial fibrillation (AF) is the most common cause of an irregular, narrow-complex tachycardia, affecting approximately 33 million people worldwide.^3^ Patients of older age are at increased risk of developing AF, as well as patients with hypertensive, valvular and ischaemic heart disease.^4^ AF can be triggered by acute alcohol intoxication, thyrotoxicosis, sepsis or dehydration.^5^ More recently, AF has been shown to be associated with obesity and obstructive sleep apnoea.^6^-^8^

**Table 1:** Diagnostic approach to irregular, narrow-complex tachycardia

*Key features*	*Atrial fibrillation*	*Atrial flutter*	*Multifocal atrial tachycardia*
Atrial wave morphology	Fibrillatory waves (f waves, irregular in morphology and amplitude)	Flutter waves (F waves, regular in morphology and amplitude)	At least three different P-wave morphologies in same lead
Atrial wave timing	Variable	Identical	Variable
Atrial wave cycle length	400–600 per min	240–360 per min	Usually < 130 per min
PR interval	No obvious PR interval	No obvious PR interval	Variable PR intervals
Ventricular (QRS) Response	Usually narrow QRS complexes, often vary in amplitude, constantly irregular RR intervals	Usually narrow QRS complexes, constant F/R Ratios	Usually narrow QRS complexes, random and constantly irregular RR intervals

**Fig. 3:**
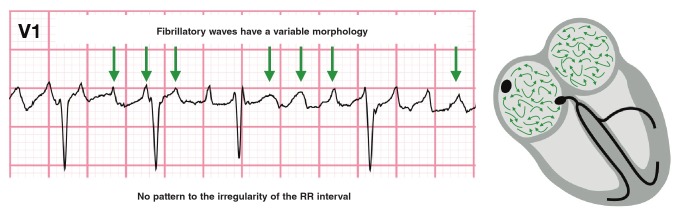
In atrial fibrillation, the chaotic atrial activity translates to irregular fibrillatory waves.

In atrial fibrillation there is chaotic, asynchronous atrial impulse propagation, with multiple wavelets that course irregularly through the atria and reach the AV node at irregular intervals, which cause irregular AV nodal conduction. On the ECG (Fig. 3), atrial fibrillation is recognised by an irregular RR interval with no pattern to the irregularity and the absence of distinct P waves. Very rapid, continuous, irregular ‘chaotic’ activity (called fibrillatory waves) can be seen. These are best seen in V1 and can be coarse or fine. Fibrillatory waves can be as fast as 400–600 per minute. The ventricular rate, however, can be fast, normal or slow, depending on AV nodal conduction.^9^

**Fig. 4:**
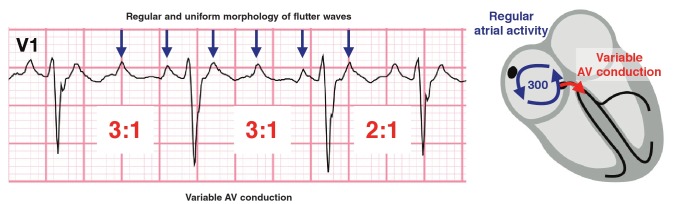
In atrial flutter with a variable block, the re-entry circuit results in uniform flutter waves.

Atrial flutter (AFL), the second most common pathological supraventricular tachyarrhythmia, shares many risk factors with atrial fibrillation.^10^ In contrast to AF, AFL is caused by rapid, continuous atrial activity around a fixed re-entry circuit, usually an anti-clockwise circuit in the right atrium. Flutter waves have a saw-tooth pattern and are best appreciated in standard lead II and lead V1 (Fig. 4). It can be difficult to differentiate atrial flutter with variable block from coarse atrial fibrillation.

In contrast to AF, flutter waves are regular and discrete, uniform in morphology (in keeping with the organised re-entry circuit) with a fixed atrial rate usually around 300 per minute (can range between 240 and 360). The ventricular rate depends on the degree of AV block (e.g. QRS rate of approximately 150 in 2:1 block, approximately 100 in 3:1 block, and approximately 75 in 4:1 block). In patients with AV node disease or who take drugs that slow AV conduction, the ventricular response to atrial flutter may be irregular, though less erratic than in atrial fibrillation.^9^,^10^

**Fig. 5:**
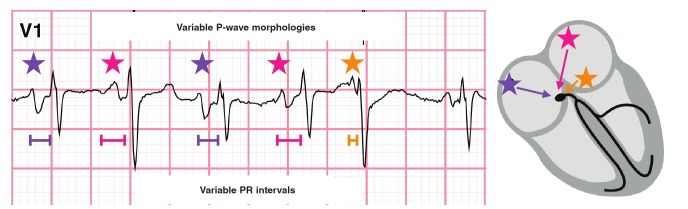
In atrial flutter with a variable block, the re-entry circuit results in uniform flutter waves.

Multifocal atrial tachycardia (MAT) is a rare condition that occurs in patients with advanced pulmonary disease or who are receiving theophylline treatment.^11^ In multifocal atrial tachycardia there is random firing of different atrial ectopic foci. MAT is defined as a rhythm with an atrial rate > 100 beats per minute with at least three morphologically distinct P waves (Fig. 5), irregular P-P intervals, and an isoelectric baseline between P waves (distinguishing MAT from AF and AFL).^9^,^10^

## The correct treatment depends on the correct diagnosis

The correct distinction between AF, AFL and MAT is of paramount importance, since the three conditions require different therapeutic approaches.

To reduce the risk of thromboembolism, appropriate oral anticoagulation should be offered to patients with AF and AFL and who do not have any contra-indications to vitamin K antagonists (VKAs) or non-vitamin K oral anticoagulants (NOACs).^2^ On the other hand, patients with MAT do not require anticoagulation.

Uncontrolled tachyarrhythmia due to atrial fibrillation or flutter can result in acute cardiac decompensation as well as tachycardia-induced cardiomyopathy (TIC) in the long term.^12^ Rate control is therefore an essential part in the treatment of AF and AFL.^13^

In atrial fibrillation, rhythm control was not found to be superior to rate control in the AFFIRM and RACE trials.^14^,^15^ Rhythm-control strategies were often associated with drug toxicity of anti-arrhythmic drugs and failure to maintain sinus rhythm in atrial fibrillation. Pulmonary vein isolation (PVI) is another rhythm-control strategy that may not require additional anti-arrhythmic drugs. Catheter ablation (pulmonary vein isolation) can improve LV systolic function in patients with AF and reduced LVEF and may improve survival.^16^

In atrial flutter, a rhythm-control strategy is often preferred over a rate-control strategy. Radiofrequency ablation is a highly effective treatment of typical atrial flutter involving the right atrial cavotricuspid isthmus. For these reasons, rate control of atrial flutter is usually not a long-term option, especially if patients are symptomatic, if the ventricular rate is difficult to control (which is not uncommon) and if there is an associated tachycardia-induced cardiomyopathy.^17^ Previous studies have found an improvement in LV systolic function after RFA in patients with AFL and a reduced LVEF.^18^,^19^

MAT is treated by treating the underlying lung disease.^20^ Betablockers are often not well tolerated in this population.
